# Prevalence and associated factors of chronic kidney disease among adults receiving antiretroviral therapy at Hawassa University Comprehensive Specialized Hospital

**DOI:** 10.1371/journal.pone.0320787

**Published:** 2025-04-03

**Authors:** Yitayew Ewnetu Mohammed, Desalegn Dawit Assele, Sinetibeb Tadesse Amsalu, Yared Habtie Tayachew, Teshome Abuka, Menbere Weldecherkos, Ashagrachew Haile, Sileshi Demelash

**Affiliations:** 1 Department of Internal Medicine, College of Medicine and Health Science, Hawassa University, Hawassa, Ethiopia; 2 Department of Public Health, College of Medicine and Health Sciences, Hawassa University, Hawassa, Ethiopia; 3 Ethiopia Public Health Institute, Addis Ababa, Ethiopia; Institute of Medicine (IOM), Maharajgunj Medical Campus, TU, NEPAL

## Abstract

**Background:**

Antiretroviral therapy (ART) improves life expectancy in people living with human immunodeficiency virus (HIV). The risk of chronic kidney disease (CKD) is greater in people living with HIV (PLWH) than in the general population, and it is becoming a significant public health issue, increasing disease progression and complicating treatment. However, patients in Africa are not routinely screened due to resource constraints, which leads to a high CKD burden. Identifying the predisposing factors is the crux of mitigating the burden of CKD. We investigate the prevalence of chronic kidney disease and associated factors among PLWH at the Hawassa University Comprehensive Specialized Hospital (HUCSH).

**Methods:**

A cross-sectional study was conducted from August 2 to September 3, 2022, at the HUCSH ART clinic, in Hawassa, Ethiopia. Data were collected from 338 PLWH through interview and medical record review. Renal function was assessed using the estimated glomerular filtration rate (eGFR) calculated using the “Chronic Kidney Disease Epidemiology Collaboration” (CKD-EPI) formula. Data were entered in Epidata version 3.01 and analyzed using Statistical Package for the Social Sciences (SPSS) version 26.0. Both bivariable and multivariable logistic regression analyses were used to identify factors associated with CKD among PLWH. An adjusted odds ratio (AOR) with a 95% Confidence Interval (CI) was reported to show the strength of the association. The goodness of fit of the model was checked by the Hosmer and Lemeshow test. The statistical significance of associations was declared at a p-value < 0.05.

**Results:**

The study included 338 PLWH with a response rate of 96%. The mean (standard deviation (SD) age of the participants was 44.4 ( ± 10.9) years. The female-to-male ratio was 1:1.8. The prevalence of CKD was 7.7% [95% CI: 5–10.7%]. History of alcohol use [AOR: 5.4; 95%CI: 1.32, 21.7], having chronic medical illness [AOR: 5.3; 95%CI: 1.45, 19.1], late stage of HIV [AOR: 5.2; 95%CI: 1.1, 25.3], opportunistic infections [AOR: 5.4; 95%CI: 1.25, 23.4], and low baseline hemoglobin level [AOR: 7.9; 95%CI: 2.58, 24.4] were significantly associated with CKD.

**Conclusion:**

The study found that CKD prevalence in PLWH was high. Factors associated with CKD include alcohol use, chronic medical illness, advanced WHO HIV stage, opportunistic infections, and low hemoglobin. Therefore, PLWH should be regularly screened for early diagnosis and management of CKD, and those with associated factors should be closely monitored.

## Introduction

Human immunodeficiency virus (HIV) associated with chronic kidney diseases (CKD) has become a major public health issue, particularly in Africa [1]. The global prevalence of CKD in people living with HIV (PLWHIV) is 6.4%, with Africa showing the highest prevalence at 7.9% [2]. In Ethiopia, the prevalence of CKD among PLWH patients ranges from 7.6% to 20.7% [3,4]. PLWH faces age-related conditions and side effects from long-term ART exposure, including CKD and cardiovascular disorders [5]. CKD is progressive and may progress to end-stage renal disease (ESRD), affecting quality of life and death risk [6].

CKD in PLWH could be caused by various factors including aging, opportunistic infections, polypharmacy, drug interactions, drug interactions, and comorbid medical conditions like hypertension and Diabetes mellitus [7,8]. HIV by itself can also lead to CKD via direct infecting of the kidneys leading to HIV-associated nephropathy (HIVAN) pathogenesis through viral gene expression and glomerular and tubular epithelial cell infection [9]. The kidney can serve as a reservoir for HIV strains, leading to dysregulation of cellular pathways, inflammation, cell death, and cytoskeletal homeostasis [10].

HIV infection and chronic kidney disease are linked, requiring careful management to address therapeutic difficulties like drug-drug interactions and toxicity [11]. The most effective strategy is suppressing HIV viral load using Highly active antiretroviral therapy (HAART), affecting patient prognosis and preventing kidney disease treatment of HIVAN in particular may require a combination of antiretroviral therapy, renin-angiotensin system inhibitors, and corticosteroids, but there are still gaps in its management [12,13].

Antiretroviral therapy has improved HIV survival, but long-term exposure can lead to age-related conditions and side effects like cardiovascular and kidney diseases, cardiovascular and kidney diseases, affecting quality of life and increasing death risk [6]. Providers should be cautious about potential nephrotoxic effects and drug-drug interactions, and adjust dosages based on estimated glomerular filtration rate (eGFR) [14]. Studies show that CKD in PLWH is influenced by various factors including direct virus insults, aging, hepatitis co-infection, prolonged ART exposure, low CD4 count, high viral load, coinfections, opportunistic infections, polypharmacy, and genetics [15–17]. Noncommunicable diseases(NCDs) like hypertension and diabetes also increase the risk of CKD [18,19], emphasizing the need for routine screening for early diagnosis and management of risk factors [20].

Early diagnosis and management of CKD in PLWH is crucial for maintaining kidney function and preventing progression to ESRD [21]. The HIV care guideline recommends routine renal function tests [22], but these are often overlooked due to resource constraints in Africa, including Ethiopia. There is a lack of data on the prevalence and associated factors of CKD among PLWH in the study setting. We investigated the prevalence of CKD and associated factors among HIV-infected people at the Hawassa University Comprehensive Specialized Hospital.

## Method

### Study design, setting, and period

A cross-sectional study was conducted at Hawassa University Comprehensive Specialized Hospital (HUCSH), Ethiopia, from August 2, 2022, to September 3, 2022. The hospital is located in Hawassa city and started healthcare delivery services in 2006. It has 400 inpatient beds and provides high-quality services for about 20 million populations in the southern regions. HUCSH also serves as a training center for medical students and health science trainees. The ART clinic at HUCSH has been providing care to 2800 adult PLWH [23].

### Population

All adult PLWH on ART and had a follow-up visit at the ART clinic between August 2, 2022, and September 3, 2022, were the study population. However, individuals who were critically ill and unable to participate in the interview, pregnant women, had solitary kidneys, and received ART for less than 6 months were excluded. In addition, individuals with incomplete laboratory and clinical data for essential variables were excluded from the study.

### Sample size determination and sampling procedure

The sample size was calculated using a single population formula by Epi info software version 7 with the assumption of: 20.7% anticipated proportion of CKD which was taken from a similar study conducted at ART clinic in Mettu Karl Referral Hospital, Mettu town, south-west Ethiopia [3], a margin of error of 0.05 at a 95% confidence level, and a 10% non-response rate, the sample size was 277. Sample size was also calculated for selected potential variables; gender, CD4 count, WHO stage, hypertension, diabetes, and viral load (Table 1). The largest calculated sample size (i.e., 320) was used to yield the maximum sample size, and the final sample size taking 10% contingency was 352.

**Table 1:** Summary of sample size calculation for associated factors with chronic kidney disease among adults receiving antiretroviral therapy at HUCSH.

Variables	CI	Power	Ratio	% of outcomes in unexposed	AOR	Sample size	Ref
Male gender	95%	80%	1	48.1	2.05	276	[24]
Low CD4 count	95%	80%	1	34	4.31	74	[3]
Advanced WHO stage	95%	80%	1	19.1	2.39	238	[3]
Hypertension	95%	80%	1	19.1	2.13	320	[3]
Diabetes	95%	80%	1	68.5	4.29	124	[3]
High viral load	95%	80%	1	40.7	3.10	118	[24]

### Sampling technique

A systematic sampling method was used to select the study participants. The average number of adult PLWH with a follow-up visit at the ART clinic per day was 30. During the data collection period, 720 adult PLWH were expected to have a follow-up visit. The sampling interval determined using the formula K =  N/n was two (720/352 =  2.04). The first patient to be interviewed was selected from the first three individuals using the lottery method. Then, every second patient was selected until the desired sample size was met.

### Study variables

The dependent variable of the study was chronic kidney disease among PLWH. The independent variables were sociodemographic factors (age, sex, marital status, residence, educational status), substance use-related factors (smoking, alcohol), and clinical factors including body mass index (BMI); family history of CKD; chronic medical illnesses (hypertension, diabetes mellitus, cardiac illness); baseline CD4 count; baseline viral load; WHO clinical stage; duration of HIV infection; hemoglobin level; opportunistic infections; treatment-related factors (ART regimen, ART duration, drug adherence, treatment failure).

### Data collection method

Data were collected using a structured questionnaire through direct patient interviews and revision of patients’ medical charts for relevant clinical and laboratory information. The questionnaire was adopted from different literature taking into account the characteristics of the study population in this study [4,15,17,24]. Blood samples were collected by trained laboratory professionals from each study participant for serum creatinine assessment. About 3–5mL of venous blood was drawn from each participant and dispensed into a gel-coated serum separator test tube and centrifuged at 3500 rpm for 10 minutes to separate the serum. The separated serum was transferred to a Nunc tube and kept frozen at − 20 ºC until processed. Serum creatinine was analyzed using a Mindray BS-230 analyzer. Renal function was assessed using the estimated glomerular filtration rate (eGFR) calculated using the “Chronic Kidney Disease Epidemiology Collaboration” (CKD-EPI) formula. Study participants’ medical record was also revised for previous serum creatinine measurements, which were used to establish chronicity in participants with renal function impairment.

### Variables definition

**CKD:** is defined in this study as the presence of either of the following findings for >  3 months [22,25].

- Decreased eGFR <  60 ml/min/1.73 m2. GFR was calculated using the CKD-EPI formula which was shown to be more accurate than the Cockcroft Gault formula and modification of diet in renal disease (MDRD) formulas as seen from studies on PLWH.
- One or more of the markers of kidney damage: Albuminuria (albumin excretion rate (AER) ≥  30 mg/24 hours; albumin to creatinine ratio (ACR) ≥  30 mg/g [ ≥ 3 mg/mmol]); Urine sediment abnormalities (red blood cell (RBC) casts, white blood cell (WBC) casts); Structural abnormalities detected by imaging (Small and hyperechoic kidneys, Renal masses or enlarged kidneys due to infiltrative diseases, Renal artery stenosis)

**Hypertension:** in this study was defined based on the previous diagnosis of hypertension or blood pressure of 140/ 90 mmHg or more on two readings taken at least 5 minutes apart [26].

**Diabetes mellitus:** defined as fasting blood sugar ≥  126 mg/dl, HgA1c ≥  6.5%, random blood sugar ≥  200 with symptoms, or a two-hour oral glucose tolerance test ≥  200mg/dl [27].

**Adherence:** adherence was calculated as the number of doses of ART taken/number of prescribed doses of ART multiplied by 100%. Good adherence, > 95%, fair adherence, 85–95%, and poor adherence, <  85% of doses taken [24].

### Data quality management

To ensure the quality of the data, before data collection, the data collectors were trained for one day by the principal investigator on the objective, relevance of the study, and confidentiality of the information. The pretest was done by taking 5% of the total sample size two weeks before the actual data collection to check its variability. The questionnaire was then assessed for its clarity, length, and completeness, and necessary corrections were made accordingly. Data collectors were trained professional nurses working in ART clinics and medical interns.

### Data management and analysis

Data was checked manually for completeness and entered into Epidata version 3.1. The processed data in the Epidata was exported to Statistical Package for the Social Sciences (SPSS) version 26.0 software for statistical analysis. Descriptive analyses were carried out, and categorical variables were summarized using frequencies and percentages while continuous variables were summarized using mean ±  standard deviation (SD). Binary logistic regression analysis was used to identify factors associated with CKD among PLWH. A variable with a p-value <  0.25 in the bivariable analysis was recruited for multi-variable analysis. An Adjusted Odds Ratio (AOR) with a 95% Confidence Interval (CI) was reported to show the strength of the association. The goodness of fit of the model was checked by the Hosmer and Lemeshow test (p = 0.268). All statistical significance of associations was declared at a p-value < 0.05.

### Ethical consideration

Ethical clearance was obtained from the Hawassa University College of Public Health and Medical Sciences Institutional Review Board. An official letter was obtained from the Department of Internal Medicine to the respective bodies of the referral hospital for their kind permission and cooperation throughout the study. Information obtained from the participant’s medical records was kept confidential by not recording participants’ names and phone numbers on questionnaires. A written consent was also taken from study participants.

## Results

### Sociodemographic characteristics

The study involved 338 people living with HIV (PLWH) with a response rate of 96%. The majority, 216 (63.9%) of participants were females. The mean (standard deviation (SD)) age of the participants was 44.4 ( ± 10.9) years, ranging from 18 to 72 years. 142 (42% of the study participants) were within the age group of 40–49 years, and 98 (29%) of the patients were older than 50 years old. During the time of the study, almost half of the patients (52.4%) were married, and 91.7% were living in urban areas (Table 2).

**Table 2:** Sociodemographic characteristics of PLWH on follow-up at HUCSH ART clinic, 2023.

Variable		Frequency(n = 338)	Percent
**Age**	18–29	29	8.6
	30–39	69	20.4
	40–49	142	42.0
	≥ 50	98	29.0
**Gender**	Male	122	36.1
	Female	216	63.9
**Marital status**	Never married	43	12.7
	Married	177	52.4
	Widowed	78	23.1
	Divorced	40	11.8
**Residence**	Urban	310	91.7
	Rural	28	8.3
**Educational status**	Unable to read and write	13	3.8
	Able to read and write	14	4.1
	Primary school	136	40.2
	Secondary or preparatory school	125	37.0
	College and above	50	14.8

### Clinical and substance use-related characteristics

The majority of the study participants (60.4%) have a body mass index (BMI) in the normal range (18.5–24.99), and 35.2% of patients have a BMI above the normal range ( > 25). Fifty-four participants (16%) have a history of chronic medical illness, the most common being hypertension (7.4%), followed by DM (4.4%). Twenty-eight participants (8.3%) have a history of smoking, and 14.2% of patients have a history of current alcohol use (Table 3).

**Table 3:** Clinical and substance use-related characteristics of PLWH on follow-up at HUCSH ART clinic, 2023.

Variable		Frequency (n = 338)	Percent
**BMI**	< 18.5	15	4.4
	18.5 – 24.99	204	60.4
	≥ 25	119	35.2
**History of Smoking**	Yes	28	8.3
	No	310	91.7
**History of Alcohol Intake**	Yes	48	14.2
	No	290	85.8
**History of Chronic Medical Illness**	Yes	54	16.0
	No	284	84.0
**Type of Chronic medical illness**	Diabetes mellites	15	4.4
	Hypertension	25	7.4
	Cardiac illness	5	1.5
	Others	9	2.7
**Duration of HIV**	< 5 years	27	8.0
	5 - 10 year	92	27.2
	> 10 years	219	64.8
**Stage of HIV at baseline**	Early stage	127	37.6
	Advanced stage	211	62.4
**ART duration**	< 5 years	30	8.9
	5 - 10 years	109	32.2
	> 10 years	199	58.9
**Current ART regimen**	1J (TDF, 3TC, DTG)	295	87.3
	2H (TDF, 3TC, ATV/r)	14	4.1
	2I (ABC, 3TC, LPV/r)	14	4.1
	2F (AZT, 3TC, ATV/r)	3	0.9
	1E (TDF, 3TC, EFV)	12	3.6
**Treatment failure**	Yes	34	10.1
	No	304	89.9
**Drug adherence to ART**	Good	325	96.2
	Fair	4	1.2
	Poor	9	2.7
**History of Opportunistic Infection**	Yes	175	51.8
	No	163	48.2
**Type of Opportunistic infection**	Protozoal	24	7.1
	Fungal	13	3.8
	Viral	32	9.5
	Tuberculosis	61	18.0
	Mixed	45	13.3
**Family history of CKD**	Yes	3	0.9
	No	335	99.1
**Baseline CD4 count**	< 200	215	63.6
	≥ 200	123	36.4
**Baseline Viral load**	Undetectable	75	22.2
	Detectable	195	57.7
	Undetermined	68	20.1
**Baseline hemoglobin level**	≥ 12	274	81.1
	< 12	64	18.9

BMI: Body mass index; ART; Antiretroviral therapy; CKD: Chronic kidney disease; TDF: Tenofovir disoproxil fumarate; 3TC: Lamivudine; DTG: Dolutegravir; ATV/r: Atazanavir/ritonavir; ABC: Abacavir; LPV/r: Lopinavir/ritonavir; AZT: Zidovudine; EFV: Efavirenz.

The mean (SD) duration of HIV diagnosis is 11.3 years ( ± 4), with a minimum of 1 year and a maximum of 23 years. The majority of the participants (62.4%) had advanced-stage disease (stages 3 and 4) at diagnosis. All participants are on ART, with a mean (SD) treatment duration of 10.6 years ( ± 3.8) since ART initiation. Two hundred ninety-five participants (87.3%) are on the 1J regimen (tenofovir (TDF), lamivudine (3TC), and dolutegravir (DTG)). Thirty-four patients (10.1%) have a history of treatment failure. Most of the participants (63.3%) have a baseline CD4 count of less than 200. About half of the participants (51.8%) have a history of opportunistic infections, the most common being tuberculosis (18%). Other opportunistic infections include protozoal (7.1%), fungal (3.8%), viral (9.5%), and mixed infections (16.9%) (Table 3).

### Prevalence of chronic kidney disease

**Fig 1:**
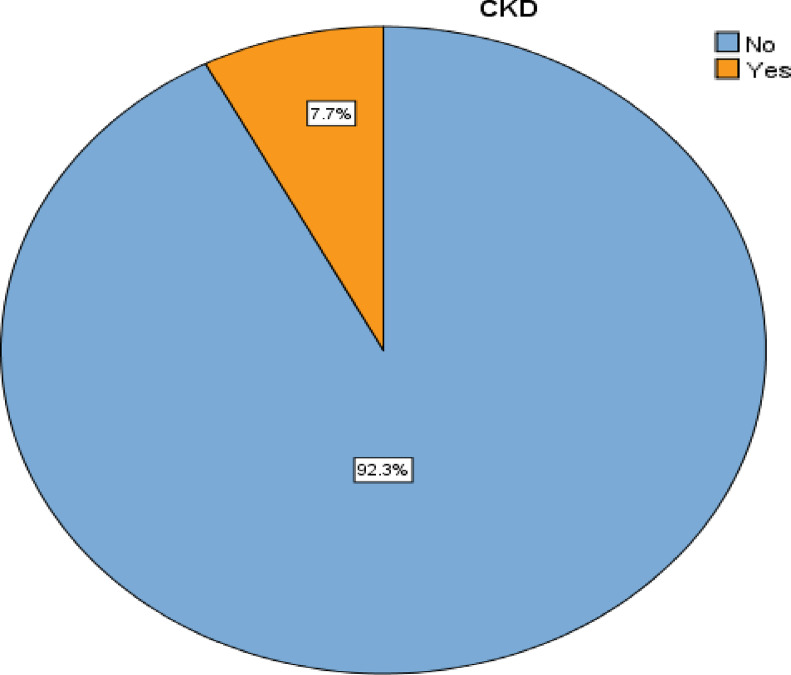
Prevalence of CKD among PLWH on follow-up at HUCSH ART clinic, 2023.

The prevalence of chronic kidney disease in this study was 7.7% [95% CI: 5–10.7%] (Fig 1). The majority of patients diagnosed with CKD (4.4%) are in the stage 3 GFR category followed by stage 2 CKD (3%).

### Associated factors for chronic kidney disease

In bivariable logistic regression analysis, BMI, duration of HIV, duration of ART, the ART regimen, history of treatment failure, drug adherence to ART, and baseline CD4 count were found to have a p-value of <  0.25. In the multivariable logistic regression model, history of current alcohol use, history of chronic medical illness, stage of HIV at baseline, history of opportunistic infections, and baseline hemoglobin level were found to have a statistically significant association with CKD.

Patients with a current history of alcohol use have a 5.5-fold higher likelihood of suffering from CKD when compared with their nonalcoholic counterparts [AOR: 5.4; 95%CI: 1.32, 21.7]. The odds of PLWH having CKD increase 5.3-fold with a history of chronic medical illness [AOR: 5.3; 95%CI: 1.45, 19.1]. PLWH with advanced stages of HIV (stages 3 and 4) at baseline have a 5.2 [AOR: 5.2; 95%CI: 1.1, 25.3] times higher risk of developing CKD than those with early stages (stages 1 and 2). PLWH who have experienced opportunistic infections have a 5.4 [AOR: 5.4; 95%CI: 1.25, 23.4] times higher likelihood of having CKD than patients who have never experienced any opportunistic infections. Moreover, PLWH with a low baseline hemoglobin level (<12 mg/dl) were about 8 [AOR: 7.9; 95%CI: 2.58, 24.4] times more likely to develop CKD as compared to their counterparts (Table 4).

**Table 4:** Binary Logistic Regression Analysis for factors associated with chronic kidney disease among PLWH at the HUCSH, 2023.

Variables		CKD		COR (95% CI)	AOR (95% CI)
		Yes (n, %)	No (n, %)		
Age	< 50	14 (5.8)	226 (94.2)	1	1
	≥ 50	12 (12.2)	86 (87.8)	2.25 (1.01, 5.06)	1.40 (0.40, 4.87)
Sex	Male	16 (13.1)	106 (95.4)	3.1 (1.36, 7.08)	1.6 (0.52, 5.1)
	Female	10 (4.6)	206 (86.9)	1	1
Smoking history	Yes	6 (6.5)	22 (93.5)	3.9 (1.44, 10.8)	1.9 (0.42, 9.2)
	No	20 (21.4)	290 (78.6)	1	1
Alcohol history	Yes	12 (25.0)	36 (75.0)	6.5 (2.82, 15.3)	5.4 (1.32, 21.7) ^*^
	No	14 (4.8)	276 (95.2)	1	1
Chronic medical illness	Yes	10 (18.5)	44 (81.5)	3.80 (1.62, 8.92)	5.3 (1.45, 19.1)
	No	16 (5.6)	268 (94.4)	1	1
WHO Stage at Baseline	Early stage	2 (1.6)	125 (98.4)	1	1
	advanced stage	24 (11.4)	187 (88.6)	8.0 (1.86, 34.5)	5.2 (1.1, 25.3) ^*^
Opportunistic infection	Yes	23 (11.1)	184 (88.9)	5.3 (1.56, 18.1)	5.4 (1.25, 23.4)
	No	3 (2.3)	128 (97.7)	1	1
Baseline viral load	Undetectable	3 (3.9)	73 (96.1)	1	1
	Detectable	19 (10.0)	171 (90.0)	2.70 (0.77, 9.41)	3.3 (0.81, 13.5)
	Undetermined	4 (5.6)	68 (94.4)	1.43 (0.30, 6.63)	1.1 (0.18, 7.25)
Baseline hemoglobin(gm/dl)	≥12	13 (4.8)	260 (95.2)	1	1
	<12	13 (20)	52 (80.0)	5.0 (2.19, 11.4)	7.9 (2.58, 24.4) ^*^

* Significant at a p-value <0.05 level; AOR: adjusted odds ratio, CI = confidence interval; COR = crude odds ratio.

## Discussion

Renal disease, including chronic kidney disease (CKD), is a common complication of HIV infection. Despite the consensus that HIV increases CKD risk, studies vary in prevalence and factors contributing to its occurrence. This study assessed the prevalence and associated factors of CKD in PLWH at HUCSH using the CKD-EPI formula, the commonest method to estimate kidney function. The study found a 7.69% prevalence of CKD, consistent with a study conducted in Jimma, Southwest Ethiopia (7.6%) [24] and Brazil (6.4%) [28].

The finding was lower than a study conducted in Gondar, northwest Ethiopia (16.1%) [24], Metu town, southwest Ethiopia (20.7%) [3], Ghana (14.5%) [29], Makurdi Nigeria (24.8%) [30], Calabar south Nigeria (15.3%) [31] and Tokyo (13%) [32]. This could be due to differences in the inclusion and exclusion criteria of the study participants, sample size, lifestyle, environmental factors, and the definition of renal impairment used. For instance, in the study conducted in Gondar, the diagnosis of CKD with parameters other than GFR such as persistent proteinuria, contributed to the higher number of CKD patients. The study done on Ghanaian HIV-positive individuals excluded patients with hypertension and diabetes mellitus, which may lead to an underestimation of the true prevalence of CKD [29].

The study reveals that PLWH with a history of chronic medical illness, such as diabetes, hypertension, or cardiac illness, are at a higher risk of developing CKD, consistent with previous studies conducted at Mettu Hospital, southwest Ethiopia [3], Uganda [33] and Nigeria [30]. The association between these illnesses and CKD may be due to their vascular effects, and they are considered traditional risk factors for the development of CKD as previous studies among non-HIV populations showed similar effects. However, further study is needed to determine whether these factors have a similar degree of effect on CKD development in both HIV and non-HIV populations, or if there is an additional risk in PLWH compared to the non-HIV population.

The study found that PLWH with advanced WHO HIV stage at baseline have a higher risk of developing CKD compared to those with early stages, consistent across studies in different parts of Ethiopia [3,4,24]. This could be due to a higher risk of opportunistic infections, kidney involvement by HIV, late presentation, and potential exposure to nephrotoxic medications among this subgroup of PLWH. Similarly, we have found that opportunistic infections (OIs) increase the risk of CKD, the finding is consistent with a study conducted at the ‘Hospital Civil de Guadalajara’ in western Mexico [34]. This could be due to direct kidney involvement or increased exposure to nephrotoxic medications. Tuberculosis, a common OI among PLWH, can cause chronic granulomas and kidney calcifications, leading to decreased glomerular filtration, and some TB medications are nephrotoxic [35,36].

This study has found that patients with low baseline hemoglobin levels are more likely to develop CKD, a finding similar to a study conducted at Mehal Meda Hospital North Shoa [37]. Hypoxic and ischemic kidney insults due to low hemoglobin may be mechanisms for CKD, but further longitudinal studies are needed to determine the cause-effect association, as CKD alone can cause significant anemia. The study revealed that HIV patients with chronic alcohol use have a higher risk of chronic kidney disease (CKD) compared to nonalcoholic, a finding also supported in a prospective, longitudinal cohort study conducted in clinical centers in the United States [38]. This is because ethanol affects the antioxidant capacity of kidneys, leading to oxidative stress that damages kidneys, but it depends on the concentration and duration of stimulation(alcohol use) [39].

This study was the first to assess the prevalence of CKD and associated factors among PLWH at HUCSH, Hawassa, Ethiopia. Therefore, the results of this study could be used as baseline data for further studies. However, the study has the following limitations. First, the cross-sectional nature of the study design limits its scope from establishing a cause-effect relationship between the identified associated factors and CKD. Second, during data collection, we found that some variables were not recorded adequately on the participant’s medical record, which led to the omission of the variables from the questionnaire and then from the analysis. Third, we have revised patients’ medical records for markers of kidney injury (albuminuria, renal imaging results, urine sediment abnormalities) to identify patients with CKD who might have been missed with sole creatinine measurement and GFR estimation. However, due to recourse limitations, results of such investigations were not available in some study participants, which could result in possible missed CKD diagnoses.

## Conclusion

In conclusion, chronic kidney disease was common in adult PLWH receiving antiretroviral therapy and follow-up care at the ART clinic in Hawassa University Comprehensive Specialized Hospital, Hawassa, Ethiopia. Alcohol use, chronic medical illness, advanced WHO stage at baseline, opportunistic infections, and low hemoglobin level were factors associated with CKD. Therefore, PLWH receiving ART should have a regular renal function assessment, and those individuals with a positive history of the factors commonly associated with CKD should be closely monitored.

## Supporting information

S1 Data Datases analyzed for this study.
(XLS)

## Abbreviations

AOR: Adjusted odds ratio
AIDS: Acquired Immunodeficiency Syndrome
ART: Antiretroviral therapy
CD4: Cluster of Differentiation 4
CKD: chronic kidney disease
GFR: Glomerular filtration rate
ESRD: end-stage renal disease
HIVAN: HIV-associated nephropathy.
